# Factors Associated With Limited Cancer Health Literacy Among Chinese People: Cross-sectional Survey Study

**DOI:** 10.2196/42666

**Published:** 2023-05-24

**Authors:** Yi Shan, Meng Ji, Zhaoquan Xing, Zhaogang Dong

**Affiliations:** 1 School of Foreign Studies Nantong University Nantong China; 2 School of Languages and Cultures University of Sydney Sydney Australia; 3 Department of Urology Qilu Hospital of Shandong University Ji'nan China; 4 Department of Clinical Laboratory Qilu Hospital of Shandong University Ji'nan China

**Keywords:** factor, limited cancer health literacy, Chinese people, logistic regression

## Abstract

**Background:**

Limited cancer health literacy may be attributed to various factors. Although these factors play decisive roles in identifying individuals with limited cancer health literacy, they have not been sufficiently investigated, especially in China. There is a pressing need to ascertain the factors that effectively identify Chinese people with poor cancer health literacy.

**Objective:**

This study aimed to identify the factor associated with limited cancer health literacy among Chinese people based on the 6-Item Cancer Health Literacy Test (CHLT-6).

**Methods:**

We first categorized Chinese study participants according to the answers provided for cancer health literacy as follows: people who provided ≤3 correct answers were labeled as having limited cancer health literacy, whereas those who provided between 4 and 6 correct answers were labeled as having adequate cancer health literacy. We then adopted logistic regression to analyze the factors that were closely related to limited cancer health literacy among at-risk study participants.

**Results:**

The logistic regression analysis identified the following factors that effectively predicted limited cancer health literacy: (1) male gender, (2) low education attainment, (3) age, (4) high levels of self-assessed general disease knowledge, (5) low levels of digital health literacy, (6) limited communicative health literacy, (7) low general health numeracy, and (8) high levels of mistrust in health authorities.

**Conclusions:**

Using regression analysis, we successfully identified 8 factors that could be used as predictors of limited cancer health literacy among Chinese populations. These findings have important clinical implications for supporting Chinese people with limited cancer health literacy through the development of more targeted health educational programs and resources that better align with their actual skill levels.

## Introduction

### Background

#### Cancer Prevalence and Mortality in China

Cancer is a leading cause of death and a major public health concern in China owing to population growth and aging, as well as sociodemographic changes in the country [1,2]. There were around 4,292,000 new cancer cases and 2,814,000 cancer-related deaths in China in 2015 [1]. China considerably aggravates the global cancer burden with its estimated 22% of global cancer cases and 27% of cancer mortality [1,3]. The 4 most prevalent cancers (ie, lung, stomach, liver, and esophageal cancers) in China [1] constitute one-third to one-half of the global cancer incidence [3,4]. These common cancers are associated with poor survival in China [1]. Actually, about 60% of cancer-related deaths can be avoided by reducing exposure to modifiable risk factors [5].

#### Significance of Cancer Literacy

Health literacy is becoming an essential factor for improving health [6,7]. Patients with low health literacy tend to lack the skills required for effectively interacting with the health system and engaging in appropriate self-care, including practical knowledge on how to take medications, and interpret labels and other health information [8]. Limited health literacy has been linked to reduced health status, increased hospitalization, nonadherence to medications, medication dosing errors, and increased mortality [9,10]. Health literacy is essential for cancer patients who need to make a complex set of medical decisions on diagnoses and treatments when facing physical and emotional distress [11,12]. Improved cancer literacy is a major focus of new public health policies based upon preventive education strategies designed to reduce the cancer burden for future generations [13]. Cancer prevention is an important topic since half of cancer-related deaths can be avoided [14]. Thus, it is essential to make people aware of the risk factors and effective prevention routines (ie, improving their cancer literacy).

#### Cancer Literacy Tools Developed and Validated

Given the extensive human suffering and other costs of cancer diagnosis and care [15], some cancer health literacy scales have been well developed and validated, including the Cultural Cancer Screening Scale [16], Cervical Cancer Literacy Assessment Tool [17], 6-Item Cancer Health Literacy Test (CHLT-6) [15], 30-Item Cancer Health Literacy Test (CHLT-30) [15], Cancer Health Literacy Test-30-Spanish (CHLT-30-DKspa) [18], Portuguese CHLT-30 [19], and Chinese CHLT-30 and CHLT-6 [20]. These measures were designed to evaluate the knowledge and skills essential for locating, comprehending, and applying information from texts and materials (prose and document literacy) and for using simple arithmetic operations (quantitative literacy) [18]. Among these tools, the CHLT-6 and CHLT-3-DKspa were intended for identifying people with limited cancer health literacy [15,18]. Specifically, the CHLT-6 consists of the 6 most informative items from the CHLT-30 that was developed after deleting 36 items through cognitive interviews and 40 items through exploratory factor analysis and content coverage analysis from the initial 112 potential test items [15]. The CHLT-6 went through psychometric testing among 1306 cancer patients and was shown to be valid for determining whether a patient has limited cancer health literacy and estimating the prevalence of limited cancer health literacy [15]. The CHLT-3-DKspa was developed through translation and cultural adaptation of the CHLT-30 and validated through psychometric testing, including reliability testing (test-retest and internal consistency) and construct validity testing [18]. However, these limited number of studies did not focus on the investigation of various factors potentially associated with limited cancer health literacy, although they described varying participant demographic characteristics, including age, gender, education, ethnic groups, marital status, occupation, income, etc.

#### Gap in the Literature

To the best of our knowledge, few studies have explored the various factors predicting cancer health literacy status. A recent study examined the roles of age, education attainment, and financial resources in predicting health literacy skills [21]. More importantly, these previous investigations did not integrate participant demographic features with other essential health literacy knowledge features and skills like self-assessed disease knowledge; functional, communicative, and critical health literacy [22]; eHealth literacy [23]; and general health numeracy [24]. These health literacy knowledge features and skills are supposed to be factors that most likely identify limited cancer health literacy. However, to our knowledge, no studies have exclusively explored these critical factors.

### Objective

This study aimed to identify the factors associated with limited cancer health literacy in the Chinese population based on the CHLT-6, by including self-assessed disease knowledge; functional, communicative, and critical health literacy [22]; eHealth literacy [23]; and general health numeracy [24] in participant demographic characteristics. This study was intended to add to the limited body of evidence that supports the need to ascertain the factors associated with limited cancer health literacy. Hopefully, the findings will help health care providers to better identify and support patients with low cancer health literacy through effective screening and targeted treatment, and help policy makers to promulgate related intervention policies through national screening, education, and training among the general public in China in order to improve the overall cancer health literacy status across the country.

## Methods

### Overview

We first designed a survey questionnaire and then categorized the study participants into varying cancer health literacy groups based on the survey data collected. Afterwards, we adopted logistic regression to analyze the factors that were closely related to limited cancer health literacy measured by the study participants’ responses.

### Questionnaire Design

To achieve our objective, we designed the questionnaire through panel discussions among all researchers. The questionnaire comprised the following 2 sections: demographics and the measure (CHLT-6). The section of population demographics covered age, gender, education, self-assessed disease knowledge, the All Aspects of Health Literacy Scale (AAHLS) [22], the eHealth Literacy Scale (eHEALS) [23], and the General Health Numeracy Test (GHNT) [24] (Multimedia Appendix 1 shows the phrasing of the questions). The measure used in the questionnaire was the CHLT-6 [15] (Multimedia Appendix 2 shows the phrasing of the 6 items).

### Measure: CHLT-6

Among the currently available tools, the Cultural Cancer Screening Scale was designed to conduct breast and cervical cancer screening among Latino and Anglo women [16], the Cervical Cancer Literacy Assessment Tool was intended for cervical cancer literacy assessments with black, Latina, and Arab women in real-world settings [17], and the CHLT-6 and CHLT-30 were tools for the measurement of cancer health literacy and identification of patients with limited cancer health literacy [15]. The CHLT-30-DKspa [18], Portuguese CHLT-30 [19], and Chinese CHLT-30 and CHLT-6 [20] were translated and adapted from the original English CHLT-6 and CHLT-30 [15] for cancer health literacy assessment among Spanish-speaking Latinos, Portuguese cancer patients, and Chinese patients, respectively. After considering all these possible options, we chose the CHLT-6 over other instruments owing to the fit between the designing purpose of this scale and the objective of our study.

The CHLT-6 (Multimedia Appendix 2) categorizes participants into 2 cancer health literacy groups (limited and adequate) [15]. This measure involves 6 items derived from the CHLT-30. These 6 items have been identified as the best at distinguishing between cancer health literacy levels (limited and adequate) among the 30 items of the CHLT-30. The CHLT-6 was designed to identify people with limited cancer health literacy rapidly [15]. It comprises invariant measurement properties between gender and ethnic groups, and it has been externally validated [15]. Besides, it merely takes less than 2 minutes to administer and score [15].

### Recruitment of Participants and Questionnaire Survey

We used randomized sampling to recruit Chinese participants from Qilu Hospital affiliated to Shandong University, China. Those included in this study satisfied the following criteria: (1) being aged 17 years or older, (2) having primary education or above to understand the questionnaire items, and (3) participating in the survey voluntarily. The questionnaire survey lasted 1 month from August 1, 2022, to August 31, 2022. The questionnaire was administered via *wenjuanxing* [25], the most popular online survey platform in China. The collected data were managed in an Excel file (Microsoft Corp).

### Categorization of Participants

We categorized Chinese study participants into 2 contrastive cancer health literacy groups: people who provided ≤3 correct answers were labeled as having limited cancer literacy, whereas those who provided between 4 and 6 correct answers were labeled as having adequate cancer health literacy.

### Logistic Regression

Logistic regression was used to analyze the factors closely associated with limited cancer health literacy among the study participants. Specifically, it was used to statistically explore relations between various factors (age, gender, education, self-assessed disease knowledge, digital health literacy, general health numeracy, and communicative health literacy determined through the demographics section of the questionnaire) and differing cancer health literacy levels (the 2 groups of limited and adequate health literacy). We used 0.3 as the threshold to divide study participants (people who provided ≤3 correct answers in the CHLT-6 were labeled as having limited cancer health literacy, and those who provided between 4 and 6 correct answers were labeled as having adequate cancer health literacy). The predicted outcome of the dependent variable was the limited cancer health literacy class. The independent variables of self-assessed disease knowledge, the AAHLS, the eHEALS, and the GHNT were measured through Likert scales based on their respective question items. In regression modeling, the reference values of the categorical predictor variables were female (gender), postgraduate (education), little (self-assessed knowledge level), often (functional, communicative, and critical health literacy items of the AAHLS), strongly disagree (eHEALS), and wrong response (GHNT). The model fit was assessed using the collinearity statistics (the tolerance and its reciprocal variance inflation factors [VIFs]) of the predictor variables.

### Ethical Considerations

This study was approved by the Ethics Review Board of Qilu Hospital of Shandong University, China (number: KYLL-202208-026). The study data were anonymous to protect the privacy and confidentiality of the study participants. Since the participants took part in the survey voluntarily for supporting and promoting academic research, no compensation was provided, as per the common practice in China.

## Results

### Descriptive Statistics

Table 1 presents the descriptive statistics of the data collected. A total of 849 people participated in the survey, including 441 (51.9%) females. The age of the respondents ranged from 17 to 68 years (mean 43.68, SD 11.37 years). The respondents had different levels of education: year 6 (140/849, 16.5%), year 9 (215/849, 25.3%), year 12 (157/849, 18.5%), diploma (156/849, 18.4%), bachelor’s degree (138/849, 16.3%), and postgraduate (43/849, 5.1%). The mean scores of the subconstructs in the AAHLS were 6.30 (SD 1.38) for functional health literacy, 5.56 (SD 1.47) for communicative health literacy, and 11.41 (SD 1.97) for critical health literacy. These mean values indicated that the participants *sometimes* relied on help to read health information, they *sometimes* knew the effective ways of communication with health providers, and they were *sometimes* critical about health information, respectively. The mean score for eHealth literacy was 22.65 (SD 4.91), implying a relatively high level of self-assessed eHealth literacy. The mean scores for the 6 questions in the GHNT were 1.53 (SD 0.50), 1.15 (SD 0.36), 1.18 (SD 0.39), 1.92 (SD 0.27), 1.86 (SD 0.35), and 1.79 (SD 0.41), respectively. The participants provided an average of 2.57 (SD 1.13) correct answers to these 6 questions. These scores suggested that a high percentage of participants answered the 6 questions in the GHNT incorrectly, especially questions 1, 4, 5, and 6. Based on our predefined participant categorization criterion, 62.0% (526/849) and 38.0% (323/849) of the participants were labeled as having limited and adequate cancer health literacy, respectively.

**Table 1:** Descriptive statistics.

Variable			Value (N=849)
Age (years), min-max; mean (SD)			17-68; 43.68 (11.37)
Female gender, n (%)			441 (51.9)
**Education, n (%)**			
	Year 6	140 (16.5)	
	Year 9	215 (25.3)	
	Year 12	157 (18.5)	
	Diploma	156 (18.4)	
	Bachelor’s degree	138 (16.3)	
	Postgraduate	43 (5.1)	
Self-Assessed Disease Knowledge^a^, min-max; mean (SD)			1-4; 2.40 (0.98)
**Functional Health Literacy Scale (FHL)^b^, min-max; mean (SD)**			
	FHL Item 1	1-3; 2.05 (0.76)	
	FHL Item 2	1-4; 2.18 (0.98)	
	FHL Item 3	1-3; 2.08 (0.75)	
	FHL_SUM	3-10; 6.30 (1.38)	
**Communicative Health Literacy Scale (COHL)^c^, min-max; mean (SD)**			
	COHL Item 1	1-3; 1.78 (0.77)	
	COHL Item 2	1-3; 1.88 (0.75)	
	COHL Item 3	1-3; 1.90 (0.75)	
	COHL_SUM	3-9; 5.56 (1.47)	
**Critical Health Literacy Scale (CRHL)^d^, min-max; mean (SD)**			
	CRHL Item 1	1-3; 1.98 (0.74)	
	CRHL Item 2	1-3; 1.94 (0.73)	
	CRHL Item 3	1-3; 1.94 (0.75)	
	CRHL Item 4	1-3; 2.00 (0.74)	
	CRHL Item 5	1-3; 1.97 (0.74)	
	CRHL Item 6	1-3; 1.58 (0.49)	
	CRHL_SUM	6-18; 11.41 (1.97)	
**Electronic Health Literacy Scale (eHEALS)^e^, min-max; mean (SD)**			
	eHEALS Item 1	1-3; 2.80 (1.20)	
	eHEALS Item 2	1-3; 2.80 (1.19)	
	eHEALS Item 3	1-3; 2.81 (1.18)	
	eHEALS Item 4	1-3; 2.92 (1.18)	
	eHEALS Item 5	1-3; 2.75 (1.22)	
	eHEALS Item 6	1-3; 2.86 (1.23)	
	eHEALS Item 7	1-3; 2.87 (1.20)	
	eHEALS Item 8	1-3; 2.84 (1.20)	
	eHEALS_SUM	8-24; 22.65 (4.91)	
**General Health Numeracy Test (GHNT)^f^, min-max; mean (SD)**			
	GHNT Item 1	1-3; 1.53 (0.50)	
	GHNT Item 2	1-3; 1.15 (0.36)	
	GHNT Item 3	1-3; 1.18 (0.39)	
	GHNT Item 4	1-3; 1.92 (0.27)	
	GHNT Item 5	1-3; 1.86 (0.35)	
	GHNT Item 6	1-3; 1.79 (0.41)	
	GHNT_number of total correct answers	0-6; 2.57 (1.13)	
Limited cancer health literacy, n (%)			526 (62.0)

^a^The Self-Assessed Disease Knowledge scale allows participants to report their general disease knowledge based on the following Likert scale: 1=very well, 2=a lot, 3=some, and 4=very little.

^b^The Functional Health Literacy Scale includes 3 items (Multimedia Appendix 1). The sum of Functional Health Literacy Scale item scores is provided.

^c^The Communicative Health Literacy Scale includes 3 items (Multimedia Appendix 1). The sum of Communicative Health Literacy Scale item scores is provided.

^d^The Critical Health Literacy Scale includes 6 items (Multimedia Appendix 1). The sum of Critical Health Literacy Scale item scores is provided.

^e^The Electronic Health Literacy Scale includes 8 items (Multimedia Appendix 1). The sum of Electronic Health Literacy Scale item scores is provided.

^f^The General Health Numeracy Test includes 6 items (Multimedia Appendix 1).

### Logistic Regression Analysis to Explore the Relations Between Predictors and Cancer Health Literacy Levels

Table 2 shows the collinearity statistics of the predictor variables, which included age, gender, educational level of the study participants, and several health literacy measures. The measures encompassed self-reported disease knowledge, sum of Functional Health Literacy Scale scores (FHL_SUM) [22], sum of Communicative Health Literacy Scale scores (COHL_SUM) [22], sum of Critical Health Literacy Scale scores (COHL_SUM) [22], sum of eHEALS scores (eHEALS_SUM) [23], and sum of GHNT scores (GHNT_SUM) [24]. The tolerance and its reciprocal VIFs of the predictor variables were all within acceptable levels (Table 2), which permitted the use of regression modeling as shown below.

Table 3 shows the results of the logistic regression modeling of the relations between the predictor variables and the 2 groups of respondents with limited and adequate health literacy. According to this table, the odds of an individual being in the limited cancer health literacy group increased significantly when the individual was male (odds ratio [OR] 1.04, 95% CI 1.03-1.05; *P*<.001) and had education of year 6 (OR 11.94, 95% CI 2.43-58.59; *P*<.001), year 9 (OR 16.09, 95% CI 3.33-77.77; *P*<.001), year 12 (OR 15.25, 95% CI 3.12-74.54; *P*<.001), diploma (OR 13.36, 95% CI 2.77-64.41; *P*<.001), or bachelor’s degree (OR 5.55, 95% CI 1.16-26.65; *P*=.003).

**Table 2:** Collinearity statistics of the predictor variables.

Predictor variable	Collinearity statistics	
	Tolerance	VIF^a^
Age	0.83	1.21
Gender	0.95	1.06
Education	0.79	1.27
Self-Assessed Disease Knowledge	0.98	1.02
FHL_SUM^b^	0.99	1.01
COHL_SUM^c^	0.85	1.18
CRHL_SUM^d^	0.93	1.07
eHEALS_SUM^e^	0.80	1.26
GHNT_SUM^f^	0.96	1.05

^a^VIF: variance inflation factor.

^b^FHL_SUM: sum of Functional Health Literacy Scale scores.

^c^COHL_SUM: sum of Communicative Health Literacy Scale scores.

^d^CRHL_SUM: sum of Critical Health Literacy Scale scores.

^e^eHEALS_SUM: sum of Electronic Health Literacy Scale scores.

^f^GHNT_SUM: sum of General Health Numeracy Test scores.

**Table 3:** Logistic regression analysis (threshold=0.3) for the predicted outcome of limited cancer health literacy.

Variable		B	SE	Wald	*df*	*P* value	Exp(B)	95% CI for Exp(B)	
								Lower	Upper
Age		0.04	0.01	38.64	1	<.001	1.04	1.03	1.05
Male gender (reference: female)		0.41	0.18	5.39	1	.02	1.51	1.07	2.13
**Education**									
	Postgraduate (reference)	N/A^a^	N/A	24.99	5	<.001	N/A	N/A	N/A
	Year 6	2.48	0.81	9.33	1	<.001	11.94	2.43	58.59
	Year 9	2.78	0.80	11.94	1	<.001	16.09	3.33	77.77
	Year 12	2.72	0.81	11.33	1	<.001	15.25	3.12	74.54
	Diploma	2.59	0.80	10.43	1	<.001	13.36	2.77	64.41
	Bachelor’s degree	1.71	0.80	4.59	1	.03	5.55	1.16	26.65
COHL_SUM^b^		0.22	0.07	11.02	1	<.001	1.24	1.09	1.42
**CRHL^c^ Item 4^d^ result**									
	Little (reference)	N/A	N/A	16.08	2	<.001	N/A	N/A	N/A
	Often	0.95	0.24	15.93	1	<.001	2.60	1.63	4.15
	Sometimes	0.50	0.21	5.64	1	.02	1.64	1.09	2.47
CRHL Item 6^d^ for education (reference: health facilities)		−0.41	0.18	5.35	1	.02	0.66	0.47	0.94
**eHEALS^e^ Item 3^f^ result**									
	Strongly agree (reference)	N/A	N/A	13.77	4	.01	N/A	N/A	N/A
	Strongly disagree	−0.45	0.42	1.14	1	.29	0.64	0.28	1.45
	Disagree	−0.43	0.38	1.25	1	.26	0.65	0.31	1.38
	Unsure	−1.06	0.35	8.99	1	<.001	0.35	0.17	0.69
	Agree	−0.82	0.37	4.77	1	.03	0.44	0.21	0.92
**eHEALS Item 4^f^ result**									
	Strongly agree (reference)	N/A	N/A	18.88	4	<.001	N/A	N/A	N/A
	Disagree	0.83	0.45	3.41	1	.06	2.29	0.95	5.51
	Unsure	−0.57	0.34	2.93	1	.09	0.56	0.29	1.09
	Agree	−0.40	0.31	1.62	1	.20	0.67	0.37	1.24
	Strongly agree	0.05	0.34	0.02	1	.89	1.05	0.54	2.04
**eHEALS Item 6^f^ result**									
	Strongly agree (reference)	N/A	N/A	11.42	4	.02	N/A	N/A	N/A
	Disagree	−1.07	0.41	6.82	1	.01	0.34	0.15	0.76
	Unsure	−1.21	0.39	9.86	1	<.001	0.30	0.14	0.63
	Agree	−1.15	0.36	10.35	1	<.001	0.32	0.16	0.64
	Strongly agree	−0.99	0.38	6.99	1	.01	0.37	0.18	0.77
eHEALS_SUM^g^		−0.09	0.03	7.89	1	<.001	0.92	0.86	0.97
GHNT^h^ Item 6^i^ for correct (reference: wrong)		−1.07	0.23	21.27	1	<.001	0.34	0.22	0.54
**Self-Assessed Disease Knowledge result**									
	Little (reference)	N/A	N/A	26.80	3	<.001	N/A	N/A	N/A
	Very well	0.10	0.26	0.16	1	.69	1.11	0.67	1.83
	Well	−0.17	0.24	0.52	1	.47	0.84	0.53	1.34
	Some	−0.80	0.23	11.90	1	<.001	0.45	0.29	0.71
Constant		0.47	1.43	0.11	1	.74	1.60	N/A	N/A

^a^N/A: not applicable.

^b^COHL_SUM: sum of Communicative Health Literacy Scale scores.

^c^CRHL: Critical Health Literacy Scale.

^d^The Critical Health Literacy Scale includes 6 items (Multimedia Appendix 1).

^e^eHEALS: Electronic Health Literacy Scale.

^f^The Electronic Health Literacy Scale includes 8 items (Multimedia Appendix 1).

^g^eHEALS_SUM: sum of Electronic Health Literacy Scale scores.

^h^GHNT: General Health Numeracy Test.

^i^The General Health Numeracy Test includes 6 items (Multimedia Appendix 1).

Interestingly, when an individual reported lower levels of self-assessed disease knowledge, the odds of that individual being in the limited cancer health literacy group decreased significantly, questioning the reliability of this self-report scale among participants (some self-assessed disease knowledge: OR 0.45, 95% CI 0.29-0.71; *P*<.001).

The odds of an individual being in the limited cancer health literacy group decreased significantly when the individual reported overall higher levels of eHealth literacy (eHEALS_SUM). We coded the responses to the 8 items of the eHEALS in an ascending order as follows: 1=strongly disagree, 2=disagree, 3=not sure, 4=agree, and 5=strongly agree. An increase in the sum score thus indicated a higher level of confidence in seeking and using online health information. The results showed that the odds of a study participant being in the limited cancer health literacy group decreased significantly when the self-reported eHealth literacy level increased (eHEALS_SUM: OR 0.92, 95% CI 0.86-0.97; *P*<.001). The eHEALS includes 8 short questions of the self-assessed ability to search, appraise, and use online health information. The results showed that although an overall higher level of self-reported eHealth literacy was a significant predictor of adequate health literacy, the interpretation of participants’ responses to individual questions in the eHEALS required greater discretion and cultural sensitivity. For example, it was found that when answering Question 3 (I know how to find useful health information on the internet), moderate levels of self-reported eHealth literacy were significant predictors of reduced odds of limited cancer health literacy (eHEALS Item 3 [agree]: OR 0.44, 95% CI 0.21-0.92; *P*=.03; eHEALS Item 3 [unsure]: OR 0.35, 95% CI 0.17-0.69; *P*<.001). The odds of being in the limited cancer health literacy group did not change significantly when the participant answered this question with higher (strongly agree) or lower levels of confidence (disagree: *P*=.26 or strongly disagree: *P*=.29).

The results also revealed the limited discrimination effect of some questions of the eHEALS among the Chinese study participants. For example, there were no significant changes among the 5 categories of eHEALS Item 4 (strongly agree, agree, unsure, strongly disagree, and disagree). Moreover, regardless of the response to eHEALS Item 6, the odds of being in the limited cancer health literacy group dropped significantly across the study population.

Limited communicative health literacy and general health numeracy were statistically significant predictors of limited cancer health literacy among the participants (COHL_SUM: OR 1.24, 95% CI 1.09-1.42; *P*<.001). A correct response to the last question of the GHNT (GHNT Item 6; regarding how to interpret breast cancer screening test results) predicted decreased odds of being in the limited cancer health literacy group (GHNT Item 6: OR 0.34, 95% CI 0.24-0.54; *P*<.001).

An important finding of this study was that a higher level of mistrust in health professionals was a significant predictor of limited cancer health literacy (“Are you the sort of person who might question your doctor’s or nurse’s advice based on your own research?”; CRHL Item 4 [often]: OR 2.6, 95% CI 1.63-4.15; *P*<.001; CRHL Item 4 [sometimes]: OR 1.64, 95% CI 1.09-2.47; *P*=.02). Since we used CRHL Item 4 (rarely) as the reference category, the results suggested that the odds of being in the limited cancer health literacy group increased significantly when study participants “often” or “sometimes” challenged health professionals when compared to those who rarely did so. The last question of the CRHL was “What do you think matters most for everyone’s health? (Tick one answer only)”: (1) information and encouragement to lead healthy lifestyles, or (2) good housing, education, decent jobs, and good local facilities. We used the second option as the reference category. It was found that among Chinese participants, the odds of being in the limited cancer health literacy group decreased significantly by 34% among those who chose the first option (CRHL Item 6 [information and lifestyles]: OR 0.66, 95% CI 0.47-0.94; *P*=.02).

**Figure 1:**
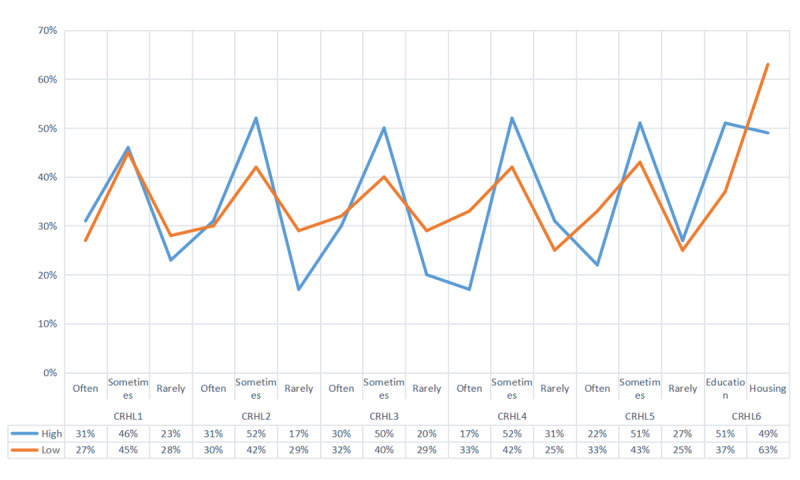
Responses to questions in the Critical Health Literacy Scale (CRHL).

Figure 1 shows the contrastive patterns of responses to questions in the CRHL among individuals with limited versus adequate cancer health literacy levels. When an individual was allocated to the limited cancer health literacy group, they tended to be less interested in seeking different kinds of health information (CRHL Item 1: preferred response was “sometimes” or “rarely”), less used to verifying whether information about their health can be trusted (CRHL Item 3: preferred response was “sometimes”), more engaged in community-level health promotion activities (CRHL Item 5: preferred response was “sometimes”), and more convinced that “good housing, education, decent jobs, and good local facilities” were more important than “information and encouragement to lead healthy lifestyles” (CRHL Item 6: preferred response was option 2 “good housing, education, decent jobs, and good local facilities”). In addition, people with limited cancer health literacy did prefer to challenge their doctor’s or nurse’s advice based on their own research (CRHL Item 4: preferred response was “often” or “sometimes”) compared to those in the reference adequate cancer health literacy group with a relatively larger proportion of people who rarely did so.

## Discussion

### Principal Findings in Relation to Relevant Studies

Limited cancer health literacy prevents patients from fully benefiting from cancer treatment, causing negative health outcomes [20]. Precisely identifying patients with limited cancer health literacy is a critical clinical challenge in China, where cancer is the leading cause of death [20]. To rise to this challenge, ascertaining the factors associated with limited cancer health literacy among Chinese people is potentially of high importance. Drawing on regression analysis of Chinese participants with varying cancer health literacy, we identified 8 factors that could be used as statistically significant predictors of limited cancer health literacy among Chinese people.

#### Principal Finding 1: The Probability of an Individual Being in the Limited Cancer Health Literacy Group Decreased Significantly When the Individual was Female

The results of our study showed that this principal finding applied to the 2-class and 3-class latent class analysis models alike. It reinforces the findings in some previous studies that females tended to have higher health literacy than males [26], and that Korean females reported significantly higher health literacy than males in understanding and filling out medical forms, understanding directions on medication bottles, and understanding written information provided by health care professionals [27]. Clouston et al reported similar findings, including that females performed better than males in health literacy tasks, and only two-fifths of females had poor health literacy compared with half of males [28]. This gap between males and females in health literacy may be attributed to the increased familiarity of females with navigating the health care system in the process of tackling health issues [27]. For example, females tended to report more health problems and have higher levels of medical service use and charges compared with males [29]. Another explanation may be related to the traditional role of females in taking care of sick family members and children [30]. This traditional gender expectation is likely to allow females to interact more with the health care system, providing them with more opportunities to build up their knowledge base, therefore leading to higher health literacy levels compared with males [27]. As such, health literacy interventions should target males with a high risk of having inadequate levels of health literacy [27].

However, this finding does not align with the finding reported by Chan et al that the limited and adequate cancer health literacy labels assigned to each class indeed refer to the same class regardless of patient gender [20]. Therefore, further research is urgently needed to ascertain the female gender as a significant predictor of adequate and high cancer health literacy or the male gender as a significant predictor of limited cancer health literacy.

#### Principal Finding 2: Low Educational Attainment was a Significant Predictor of a Participant Being in the Limited Cancer Health Literacy Group

This principal finding aligns well with the finding of Lee et al, who reported that a higher health literacy status was associated with a higher education attainment [27]. By contrast, health literacy tends to be lower in populations with lower education and older age [31,32], and lower education can directly predict poor health literacy [28]. The role of education was additionally cited by Paasche-Orlow et al, who found that the level of education was consistently associated with the level of health literacy [33]. As such, lower education attainment could be a significant factor effectively predicting limited cancer health literacy. However, it may be necessary to point out that health care professionals should not assume that patients’ health literacy is based on their education level, but provide gender-specific and tailored verbal and nonverbal communication to each patient according to their individual level of health literacy, as proposed by Lee et al [27].

#### Principal Finding 3: Lower Levels of Self-assessed Disease Knowledge Predicted a Significant Decrease in the Odds of an Individual Being in the Limited Cancer Health Literacy Group

Health literacy has been expanded to include knowledge about health management and care pathway navigation specific to diseases [15,34]. Interestingly, when an individual reported lower levels of self-assessed disease knowledge, the odds of that individual being in the limited cancer health literacy group decreased significantly in our study. It could be counter-intuitive, because it is generally assumed that lower levels of self-assessed disease knowledge are correlated with lower levels of cancer health literacy. This assumption has been confirmed in previous studies. For example, Berkman et al found that limited health literacy was related to less health knowledge [35]. Similarly, Gazmararian et al discovered that health literacy was independently associated with disease knowledge [36]. In the same vein, patients with low health literacy skills have less knowledge of their diseases and the treatments, as well as fewer correct self-management skills compared with literate patients [37,38].

Considering the previous findings, we need to further verify our counter-intuitive finding concerning self-assessed disease knowledge and ascertain the underlying causes in future studies on the one hand, and seek possible clinical explanations on the other hand. As reported in a very recent study, although patients self-assessed their disease knowledge as high and those who attended outpatient clinics or were hospitalized in wards claimed to know much about specific diseases, they actually had very little knowledge, and worse still, much of their claimed knowledge was incorrect [39]. Similarly, Lorini et al reported that what people think they know does not always equal what they really know [21]. People tend to be overconfident (they think they know more than they really know) or underconfident (they think they know less than they really know) [21]. Clinically, 2 researchers of our study (ZD and ZX), who are medical professionals, also reported such cases to our research team, and they attributed such claimed higher levels but actual lower levels of disease knowledge to patients’ inability to choose correct health knowledge when being exposed to various sources of health knowledge, especially misconceptions or myths about diseases. These findings might justify why our study participants with higher levels of self-assessed disease knowledge were classified into the limited cancer health literacy group, while study participants with lower levels of self-assessed disease knowledge were classified into the adequate cancer health literacy group.

#### Principal Finding 4: Age was Associated With Limited Cancer Health Literacy

Many existing studies found that age is a significant predictor of health literacy. Lorini et al found that health literacy levels were significantly associated with age classes (ie, the proportion of people with low health literacy increased with age) [21]. Kobayashi et al reported a similar result (ie, heath literacy was correlated with age) [40]. Specifically, they found an association between increasing age and declining health literacy [40,41]. This association can be explained by cognitive aging [40]. Some longitudinal studies showed that fluid cognitive abilities tend to decline beginning in early to mid-adulthood [42,43]. Cognitive dysfunction explained the association between increasing age and poorer performance in health literacy tests [41]. The results of our study also demonstrated that the possibility of a participant being in the limited cancer health literacy group increased significantly when the participant was older.

#### Principal Finding 5: Higher Levels of eHealth Literacy Predicted a Significant Decrease in the Odds of an Individual Being in the Limited Cancer Health Literacy Group

With the growing adoption of eHealth services, people are increasingly expected to engage in appropriate self-care and self-management of their health conditions through eHealth [44]. eHealth literacy consists of a set of skills and knowledge essential for productive interactions with technology-based health tools [45]. People with limited health literacy find it difficult to effectively use and interact with eHealth [46,47]. Based on this reasoning, we could assume that people who can use and interact with eHealth effectively may have a higher probability of having adequate or high health literacy. Individuals with higher levels of eHealth literacy could be more likely to make full use of “health services and information delivered or enhanced through the internet and related technologies” [48], including patient education, remote monitoring, communication and training, disease and outbreak tracking, and support for diagnosis and treatment decisions [49-51]. As such, several studies [44-51] support our finding that the odds of a study participant being in the limited cancer health literacy group decreased significantly when the self-reported eHealth literacy level increased.

#### Principal Finding 6: Limited Communicative (Interactive) Health Literacy Significantly Predicted a Participant’s Limited Cancer Health Literacy

Health literacy skills include the following 3 subsets of skills: (1) functional: practically applying the literacy skills needed to function effectively in daily circumstances; (2) interactive: cognitive and literacy skills used to actively participate in daily activities and apply new information to changing situations; and (3) critical: cognitive skills used to critically analyze information and impose better control over life events and situations [52]. All these abilities allow an individual to navigate within the 3 domains of health care, disease prevention, and health promotion [53]. It is more likely for adults with higher health literacy to seek health information from the internet and their health care provider, as reported in existing literature [54,55]. As a result, individuals with higher health literacy tend to have a higher probability of possessing adequate or high cancer health literacy. On the contrary, people with limited health literacy are more likely to have a higher probability of possessing limited cancer health literacy, as reported in our study. This association of health literacy with cancer-related attitudes, knowledge, and behaviors has been investigated in relevant studies [34,56,57].

#### Principal Finding 7: Lower General Health Numeracy Significantly Predicted a Participant’s Limited Cancer Health Literacy

Some studies have documented the significance of numeracy in health. Schwartz et al discovered that participants’ numeracy skills were closely related to the accuracy of applying quantitative information about the benefit of mammography to the perceived risk of death [58]. A similar study found that higher numeracy was correlated with a more consistent interpretation of breast cancer risks [59]. The importance of numeracy in the health domain was also reported by Schapira et al, who revealed that higher numeracy was associated with an improved ability to interpret treatment benefits [59], and by Estrada et al, who found that patients’ inability to handle basic probability and numerical concepts was associated with poor anticoagulation control [60]. Actually, various health-related tasks, including reading food labels, refilling prescriptions, measuring medications, interpreting blood sugar data or other clinical data, and understanding health risks, depend on numeracy skills [61]. In the CHLT-6, Items 1 and 6 are related to numeracy (ie, interpreting clinical data). We found that the majority of participants allocated to the limited cancer health literacy group answered these 2 items incorrectly. This result, coupled with the findings of relevant studies [58-61], could prove the predictive role of general health numeracy identified in our study.

#### Principal Finding 8: Mistrust in Health Professionals Predicted Limited Cancer Health Literacy

An important finding of this study was that a higher level of mistrust in health professionals was a significant predictor of limited cancer health literacy. The odds of being in the limited cancer health literacy group increased significantly when study participants *often* or *sometimes* challenged health professionals when compared with those who rarely did so. Moreover, the odds of being in the limited cancer health literacy group decreased significantly by 34% among those who chose the “information and encouragement to lead healthy lifestyles” option in CRHL Item 6.

The association between mistrust in health professionals and limited cancer health literacy may be explained by the patients’ claimed higher levels of disease knowledge. Diversified sources of health information that is not evidence-based, particularly web-based misinformation or myths about health and disease, might have misled our study participants. In China, over 80% of individuals experiencing a specific disease have sought web-based information about their condition [62]. In such a context, it is easy for them to believe web-based health information and even misinformation. This is because misinformation, not being constrained by reality, can be made more appealing, attention-grabbing, and memorable than true information [63]. Having such information in mind, people may even tend to mistrust health professionals. Confused by such information, people are likely to have low health literacy, especially as a large proportion of Chinese people tend to have critical health literacy, as identified by Shan et al [64].

### Implications

This study may add to the limited body of evidence that supports the need to ascertain the factors associated with limited cancer health literacy. The findings have important implications for medical education and training, health policy, research, and practice. Given that the majority of study participants were classified into the limited cancer health literacy group, related education and training should be conducted among the general public in China to improve the overall cancer health literacy status across the country. Screening of the most prevalent cancers needs to be carried out regularly, because there is a high prevalence of cancer in China, and a high proportion of the population with limited cancer health literacy is likely to fall victim to cancer. Furthermore, researchers can draw on the methodology and results of this study to identify more factors related to limited cancer health literacy in the Chinese population or different factors in other sociocultural and ethnic groups. As such, fresh insights could be provided into the significant predictors of limited cancer health literacy. In terms of clinical practice, the factors identified in this study could have important implications for identifying people with limited cancer health literacy and even those at risk in high cancer health literacy populations.

### Limitations

This study has some limitations. First, the data collected for this study cannot be considered representative of the overall Chinese population, since we only recruited participants from a single hospital in Shandong Province, China. The study participants may represent people in Shandong Province, a highly populated province in middle east China having relatively low socioeconomic development, but not necessarily people in the whole of China. As a result, the generalizability of the study results and principal findings to Chinese populations may be limited to some extent. Second, we could not explain some principal findings convincingly in this study (eg, principal finding 3) owing to the limited number of relevant previous studies that we could identify in the existing literature. Third, there was an issue regarding principal finding 8. Higher critical health literacy has been measured by an increase in the frequency of patients questioning the validity of health advice and recommendations given by health professionals. However, our study found that a higher level of engagement with health professionals in discussions of their health advice was a significant predictor of limited cancer health literacy among the study participants. Further research needs to be conducted to ascertain whether this finding is widely present among Chinese patients and determine the implications of defining and assessing critical health literacy in a culturally more sensitive way among Chinese populations given their traditional health cultures and the relationships between patients and health authorities.

### Conclusions

By performing regression analysis on Chinese study participants with varying cancer health literacy, we successfully identified 8 factors that could be used as predictors of limited cancer health literacy among Chinese populations. These findings have important clinical implications for identifying those with limited cancer health literacy and developing more targeted cancer educational programs and resources.

## Appendix

Multimedia Appendix 1 Items of the scales used in the study.

Multimedia Appendix 2 Translation of the 6-Item Cancer Health Literacy Test (CHLT-6) to Mandarin Chinese.

## Abbreviations

AAHLS: All Aspects of Health Literacy Scale
CHLT-6: 6-Item Cancer Health Literacy Test
CHLT-30: 30-Item Cancer Health Literacy Test
CHLT-30-DKspa: Cancer Health Literacy Test-30-Spanish
CRHL: Critical Health Literacy Scale
eHEALS: Electronic Health Literacy Scale
GHNT: General Health Numeracy Test
OR: odds ratio
SUM: sum of item scores
VIF: variance inflation factor
