# Supplementary material for: Factors Associated With Limited Cancer Health Literacy Among Chinese People: Cross-sectional Survey Study
Source: JMIR Form Res. 2023 May 24;7:e42666. doi: 10.2196/42666 (PMC10248776; doi:10.2196/42666)
Supplement: Multimedia Appendix 1 [file formative_v7i1e42666_app1.docx]

**Multimedia Appendix 1.** Items of the scales used in the study.

***Functional Health Literacy Scale***

The Functional Health Literacy Scale includes the following items:

- Item 1: How often do you need someone to help you when you are given information to read by your doctor, nurse, or pharmacist?
- Item 2: When you need help, can you easily get hold of someone to assist you?
- Item 3: Do you need help to fill in official documents?

***Communicative Health Literacy Scale***

The Communicative Health Literacy Scale includes the following items:

- Item 1: When you talk to a doctor or nurse, do you give them all the information they need to help you?
- Item 2: When you talk to a doctor or nurse, do you ask the questions you need to ask?
- Item 3: When you talk to a doctor or nurse, do you make sure they explain anything that you do not understand?

***Critical Health Literacy Scale***

The Critical Health Literacy Scale includes the following items:

- Item 1: Are you someone who likes to find out lots of different information about your health?
- Item 2: How often do you think carefully about whether health information makes sense in your particular situation?
- Item 3: How often do you try to work out whether information about your health can be trusted?
- Item 4: Are you the sort of person who might question your doctor or nurse’s advice based on your own research?
- Item 5: Do you think that there plenty of ways to have a say in what the government does about health?
- Item 6: What do you think matters most for everyone’s health? a) information and encouragement to lead healthy lifestyles; b) good housing, education, decent jobs and good local facilities.

***Electronic Health Literacy Scale***

The Electronic Health Literacy Scale includes the following items:

- Item 1: I know what health resources are available on the internet.
- Item 2: I know where to find helpful health resources on the internet.
- Item 3: I know how to find helpful health resources on the internet.
- Item 4: I know how to use the internet to answer my health questions.
- Item 5: I know how to use the health information I find on the internet to help me.
- Item 6: I have the skills I need to evaluate the health resources I find on the internet.
- Item 7: I can tell high quality from low quality health resources on the internet.
- Item 8: I feel confident in using information from the internet to make health decisions.

***General Health Numeracy Test***

The General Health Numeracy Test includes the following items:

- Item 1: Call your doctor if you have a temperature of 100.4 ºF or greater. The thermometer looks like the following: 100.2 ºF Do you call the doctor?
- Item 2: If 4 people out of 20 have a chance of getting a cold, what would be the risk of getting a cold?
- Item 3: Suppose that the maximum heart rate for a 60 year old woman is 160 beats per minute and that she is told to exercise at 80% of her maximum heart rate. What is 80% of that woman’s maximum heart rate?
- Item 4: You ate half the container of carrots. How many grams of carbohydrates did you eat?
- Item 5: Your doctor tells you that you have high cholesterol. He informs you that you have a 10% risk of having a heart attack in the next 5 years. If you start on a cholesterol-lowering drug, you can reduce your risk by 30%. What is your 5-year risk if you take the drug?
- Item 6: A mammogram is used to screen women for breast cancer. False positives are tests that incorrectly show a positive result. 85% of positive mammograms are actually false positives. If 1000 women receive mammograms, and 200 are told there is an abnormal finding, how many women are likely to actually have breast cancer?
