# Supplementary material for: Factors Associated With Limited Cancer Health Literacy Among Chinese People: Cross-sectional Survey Study
Source: JMIR Form Res. 2023 May 24;7:e42666. doi: 10.2196/42666 (PMC10248776; doi:10.2196/42666)
Supplement: Multimedia Appendix 2 [file formative_v7i1e42666_app2.docx]

**Multimedia Appendix 2.** Translation of the 6-Item Cancer Health Literacy Test (CHLT-6) to Mandarin Chinese.

| **Original CHLT6 Items** | **Chinese Translations** |
| --- | --- |
| 1. The normal range for hemoglobin for a male is 13.3–17.2 g/dl. Joe’s hemoglobin is 9.7 g/dl. Is Joe within the normal range? Yes No | 1.男性血红蛋白的正常范围是13.3-17.2 克/分升。老张的血红蛋白是 9.7克/分升。老张的血红蛋白在正常范围内吗？a. 在 b. 不在 |
| 2. A biopsy of a tumor is done to … remove it Diagnose it | 2. 对肿瘤进行活检（从身体取细胞或组织进行检验）的目的是_____。a. 切除肿瘤 b. 诊断肿瘤 c. 治疗肿瘤 |
| 3. If a patient has stage 1 cancer, it means the cancer is …Localized, In nearby organs, In distant sites | 3. 如果病人的癌症处于1期，说明癌症______。a. 癌症位于局部 b. 扩散到邻近器官 c. 已经向远处转移 |
| 4. The role of a physical therapist is to talk to a patient about emotional needs. | 4.判断对错：理疗师的工作是解决病人的情感需求。a. 对 b. 错 |
| 5. A tumor is considered “inoperable” when it cannot be treated with …Radiation therapy or Surgery or Chemotherapy | 5.不能用______治疗的肿瘤被认为是 “不能开刀的” 。a. 放疗 b. 手术 c. 化疗 |
| 6. Sally will get radiation therapy once a day, Monday through Friday. If Sally has therapy for 4 weeks, how many times will she get radiation therapy? | 6.小王星期一到星期五每天放疗一次，他4周总共放疗多少次？a. 5 b. 15 c. 20 |
